
JOURNAL INFORMATION
==============================
NLM Title Abbreviation: FEBS Open Bio
Journal ID: FEBS Open Bio
NLM Journal ID: 101580716
Journal ID: FEB4

FEBS Open Bio

EISSN: 2211-5463
Publisher: Wiley

ARTICLE INFORMATION
==============================
PMCID: PMC12956480
DOI: 10.1002/2211-5463.70203
Article ID: FEB470203
Article version: 1
Subjects: Front Cover, Front Cover

Front Cover

Electronic publication date: 2026 Mar 3
Publication date: 2026 Mar
Volume: 16
Issue: Suppl 1
Issue title: Supplement: FEBS‐IUBMB‐ENABLE 4th International Molecular Biosciences, PhD and Postdoc Conference BRIDGING MINDS, Interdisciplinary research for the future of life sciences, 10th–12th September 2025, CRUK Scotland Institute, Glasgow, United Kingdom
First page: S1
Last page: S1
Copyright: © 2026 The Authors. FEBS Open Bio published by John Wiley & Sons Ltd on behalf of Federation of European Biochemical Societies.
License: This is an open access article under the terms of the http://creativecommons.org/licenses/by/4.0/ License, which permits use, distribution and reproduction in any medium, provided the original work is properly cited.
License URL: https://creativecommons.org/licenses/by/4.0/

Figure count: 0
Table count: 0
Page count: 3

source-schema-version-number: 2.0
cover-date: March 2026
details-of-publishers-convertor: Converter:WILEY_ML3GV2_TO_JATSPMC version:6.7.1 mode:remove_FC converted:03.03.2026

==============================

### FEBS openbio

Volume 16 Supplement 1
March 2026
ISSN 2211-5463

### FEBS-IUBMB-enable Conference

4th International Molecular Biosciences
PhD and Postdoc Conference

**Glasgow 2025**

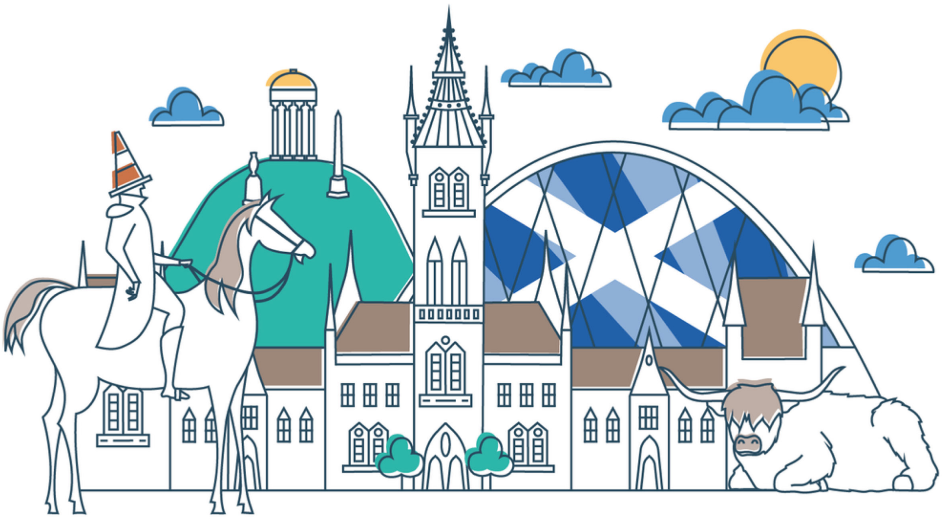

### Bridging Minds

*Interdisciplinary research for the future of life sciences*

**10th-12th September 2025**

CRUK Scotland Institute, Glasgow

WILEY

febsopenbio.org

FEBS PRESS
science publishing by scientists
